# Abdominal wall implantation of hepatocellular carcinoma

**DOI:** 10.1186/1477-7819-4-72

**Published:** 2006-10-08

**Authors:** Ali Aldahham, Shurooq Boodai, Adel Alfuderi, Ahmad Almosawi, Sami Asfer

**Affiliations:** 1 The Liver Unit, Departments of Surgery, Mubarak Al-Kabeer Hospital, Kuwait; 2 Department of Pathology, Mubarak Al-Kabeer Hospital, Kuwait; 3 Department of Surgery, Faculty of Medicine, P.O.Box 24923, Safat-13110, Kuwait

## Abstract

**Background:**

Percutaneous fine needle aspiration cytology (FNAC) became a popular method for diagnosis of hepatic masses. Abdominal wall implantation from FNAC is rare.

**Case presentation:**

We report a female patient who presented with a right upper abdominal wall mass 3 years following a fine needle aspiration cytology (FNAC) and resection of a solitary hepatocellular carcinoma (HCC) from the liver. The mass proved to be a metastatic HCC; it was locally resected with safety margins. To date (20 months later) she remains well with no recurrence.

**Conclusion:**

Implantation of tumor cells after FNAC for HCC is rare, but can happen. The availability of dynamic imaging of the liver should reduce the need for this technique in the diagnostic workup of patients suspected of having HCC

## Background

Percutaneous fine needle aspiration cytology (FNAC) became a popular method for diagnosis of hepatic masses. The reported mortality rate following this procedure ranges between 0.006% and 0.03%. The associated morbidity is 0.05% – 0.18%, this includes: hemoperitoneum, hematoma, local infection, bile peritonitis and anaphylactic shock [1]. In this report we present a rare case of abdominal wall implantation of hepatocellular carcinoma 3 years following FNAC and resection of the primary lesion.

## Case presentation

A 61-year-old female, known to suffer from chronic hepatitis C (Child Pugh A), presented in Jan 2001 to another hospital complaining of abdominal pain. CT-Scan of the abdomen showed a solitary 5 × 5 cm mass in segment VII of the liver. Fine needle aspiration cytology (FNAC) showed features consistent with HCC. AFP level at the time was normal 2.23 ng/ml (normal < 5.6 ng/ml). She traveled abroad (outside Kuwait), underwent resection of the tumor and returned back to Kuwait. A brief report stated that the tumor was resected from segment VII with about 1.5 cm safety margin with an uneventful postoperative course. Histopathology of the original tumor proved to be a well differentiated HCC with clear margins and no vascular invasion.

Upon return to Kuwait, she was under the care of the hepatologist who treating her chronic hepatitis with Pegelated Interferon and Ribaverin for 6 months. Unfortunately she remained HCV positive.

**Figure 1:**
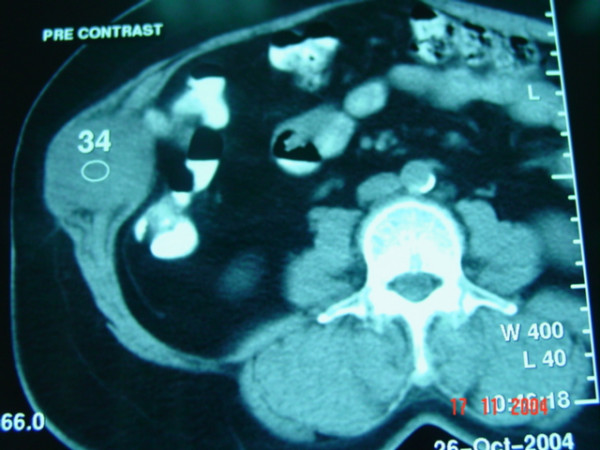
CT-Scan of the abdominal wall, shows the the mass within the abdominal wall muscles in the right hypochondrium with no intrperitoneal extension.

**Figure 2:**
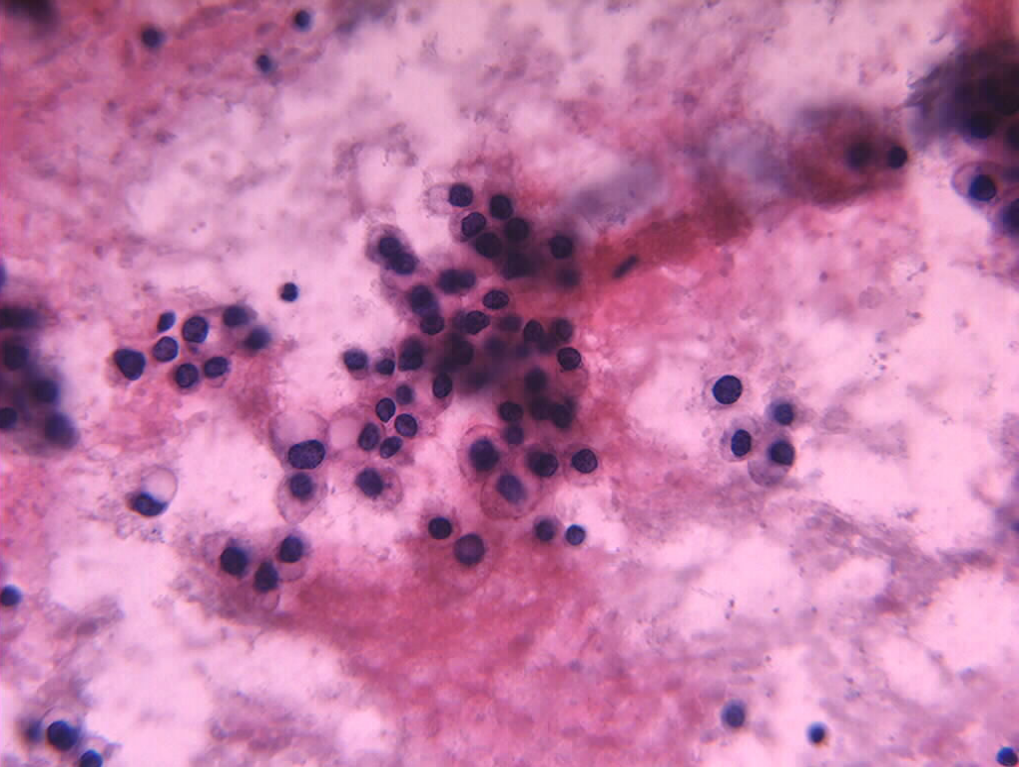
FNAC (Fine Needle Aspiration Cytology) from the mass shows polygonal cells forming microacini at places. Features consistent with Hepatocellular Carcinoma. (Papanicolaou Stain × 400)

**Figure 3:**
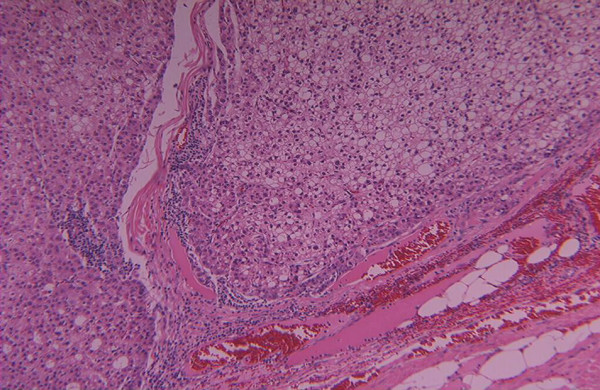
Histopathological examination of the excised mass. Shows that the tumour is made of thick hepatocyte cell plates with foci of acinar formation from the margins. (H&E stain, 10× HPF)

In Oct. 2004, three years following her original surgery, she was referred to the Liver Unit, Mubarak Al-Kabeer Hospital with an abdominal wall mass of one-year duration. On physical examination an obvious 7 × 5 cm smooth oval mass was seen in the right upper quadrant at the lateral border of her previous right subcostal scar. Clinically the mass seemed to be within the abdominal wall and the skin over it was free. CT scan of the abdomen showed a round well defined enhancing soft tissue density mass measuring 11 × 5 × 5 cm within the anterior abdominal wall with no intra-abdominal extension or skin infiltration (Figure 1). The liver was free except from signs of previous surgery in segment VII. Tumor markers were: AFP 6.23 ng/ml (normal < 5.6 ng/ml), CEA 1.5 ng/ml (normal < 6.9 ng/ml) and CA19-9 15.9 (normal < 43 ng/dl). FNAC from this mass, was consistent with metastatic HCC (Figure 2). *En-bloc *resection of the mass (including the mass, overlying skin, abdominal wall muscles and peritoneum) was performed under general anesthesia with primary closure. Histopathology of the removed specimen confirmed implantation of HCC in the abdominal muscles with free surgical margin and no peritoneal penetration (Figure 3). The patient had an uneventful recovery and was discharged home on the 7^th ^postoperative day.

To date, 20 months since resection of the metastatic mass, she remains well with normal AFP and liver functions and no evidence of recurrence in the liver or abdominal wall.

## Discussion

Implantation of hepatocellular carcinoma to the abdominal wall after percutaneous fine needle aspiration cytology is rare ranging from 1.4% to 3.4% [1]. The reported risk factors for tract implantation after FNAC include: the size of the tumor, degree of histological differentiation of HCC, a thin layer of liver parenchyma along the needle tract and the number of needle passes [1-3]. It has been shown that repeated attempts with the needle during FNAC are associated with higher chance of implantation to the abdominal wall [1]. However, implantation was reported even after a single pass [4]. There seems to be a greater chance of implantation with end-cutting biopsy needles [5].

Most HCC patients present with large and poorly differentiated tumors; they have a short survival time. Whereas patients with small, resectable and well differentiated tumors live longer and hence the probability of detecting tumor implantation in the abdominal wall after FNAC is perhaps higher in this group. The patient presented in this report had a solitary small well differentiated tumor and is still alive 5 years since her liver tumor was resected with normal liver function and no recurrence.

The tumor implantation reported in this patient, may be due to FNAC or perhaps due to spillage from the tumor during surgery. We do not have the exact details of the surgical procedure as it was done in a different country. However, the brief operative report presented to us did not indicate any surgical mishaps.

Malignant cell seeding is a well known complication of diagnostic and therapeutic procedures in HCC [6]. Denton et al indicated that there are no prospective studies about this issue; there is only small series and case reports in the literature [7]. They indicate that there is an underestimation of the risk of tumor implantation in HCC and stated that many are not diagnosed or even not reported [7].

The recent consensus meeting of the EASL(European Association for the study of the liver) and the AASLD (American association for the surgery of liver disease) in Barcelona 2005 indicated that a diagnosis of HCC in the setting of cirrhosis can confidently be made by two dynamic imaging techniques if both show increased vascularity characteristic of HCC. The panel accepted the concept of "washout" in the portal venous phase of a CT-Scan as specific for HCC; this is shown as hypo-attenuation in the late portal phase of a liver lesion which was hypervascular in the hepatic arterial phase [8]. In addition the panel agreed that an AFP of > 400 ng/ml together with only one typical imaging technique is specific for HCC [8]. Therefore, the introduction of dynamic liver studies by multidetector CT-Scan or MRI would allow clinicians to diagnose most cases of HCC with confidence without FNAC. However few atypical cases do exist and these would definitely require FNAC. The policy adopted in our Liver Unit is in accordance with the 2005 Barcelona criteria.

## Conclusion

Implantation of tumor cells after FNAC for HCC is rare, but can happen. The availability of dynamic imaging of the liver should reduce the need for this technique in the diagnostic workup of patients suspected of having HCC.

## Competing interests

The author(s) declare that they have no competing interests.

## Authors' contributions

**AAk**: Study design, data collection, data analysis and manuscript drafting. **SB**: Histopathology studies and final reporting. **AA**: Literature review and manuscript drafting. **AAf: **Data collection and literature review. **SA**: Idea originator, Critical reviewing of manuscript and final writing

All authors have read and approved the manuscript
